# The Parents' Dental Guide, &c.

**Published:** 1834-04-01

**Authors:** 


*The Parents' Dental Guide, &c.* By WM. IMRIE, Surgeon-Dentist.—London, 1834. 8vo. pp. 118.

MR. IMRIE hints pretty broadly that medical men know very little about the teeth, and he is not far wrong; but whose fault is this?—The dentists': for they lock up what they know in their own bosoms, and talk as mysteriously as a patentee, or an Egyptian priest. In the present work, however, Mr. Imrie makes no attempt to clear up the doubts of us, his erring brethren, (or rather cousins, perhaps,) but confines himself to the instruction of unprofessional parents. He conducts his pupils very tolerably to the vestibule: we hope that at some future time he will lead us into the temple.

* "During the last few days I have witnessed a case of affection of the nerves of the face, which illustrates this question of the sensibility to light in a very satisfactory manner. The mouth and tongue were drawn to the left side; there was diminished sensibility of the right side of the face and mouth, with loss of taste; the eyelids of the right side could not be closed, and did not wink in concert with the opposite: vision was perfect, but, on placing this eye close to a strong gas-light, no uneasiness was felt, and no attempt at winking followed. The sound eye could not bear the same light an instant. This case was also seen by my friend, Dr. Kay, who observed all these phenomena."

